# Associations of abdominal discomfort and length of clinical signs with surgical procedure in 181 cases of canine small intestinal foreign body obstruction

**DOI:** 10.1002/vms3.1045

**Published:** 2023-01-12

**Authors:** Alexander Chase Schoelkopf, Samuel D. Stewart, Sue A. Casale, Katy J. Fryer

**Affiliations:** ^1^ Ethos Veterinary Health Woburn Massachusetts USA; ^2^ Ethos Discovery San Diego California USA; ^3^ Surgery Department Angell Animal Medical Center Boston Massachusetts USA

**Keywords:** emergency surgery, enterotomy, gastrointestinal surgery, gastrotomy

## Abstract

**Background:**

Gastrointestinal foreign bodies are a common indication for abdominal exploratory surgery.

**Objectives:**

The objective of this study was to evaluate the relationship of pre‐operative abdominal discomfort and duration of clinical signs with surgical resolution of canine small intestinal foreign body obstructions (SIFBO).

**Methods:**

We performed a retrospective study of 181 canine abdominal exploratory surgeries for confirmed SIFBO at two referral hospitals. Animals were categorized into five surgical groups (gastrotomy after manipulation into the stomach, enterotomy, resection‐and‐anastomosis [R&A], manipulated into colon, already in colon) and further grouped by whether entry into the gastrointestinal tract (GIT) was required.

**Results:**

Abdominal discomfort was noted in 107/181 cases (59.1%), but no significant differences in abdominal discomfort rates were present among the surgical groups or between GIT entry and no entry groups. Clinical sign duration was associated with surgical procedure; median durations were R&A = 3 days (range, 1–9), enterotomy = 2 days (range, 1–14), gastrotomy = 2 days (range, 1–6), already in colon = 1.5 days (range, 1–2), and manipulated into colon = 1 day (range, 1–7). In a pairwise comparison, differences in the duration of clinical signs were found for obstructions manipulated into the colon versus R&A, gastrotomy versus R&A, and in colon versus R&A. When patients were grouped according to GIT entry, cases with entry had a longer duration of clinical signs (median = 2 days [range, 1–14] versus 1 day [range, 1–7], respectively).

**Conclusions:**

Abdominal discomfort was not associated with surgical complexity; however, the duration of clinical signs was associated with surgical complexity, with longer duration being associated with entry into the GIT and R&A. Despite statistical significance, the maximum difference of 2 days between surgical groups is unlikely to be clinically relevant.

## Graphical abstract

181 canine small intestinal foreign body surgeries were retrospectively evaluated for associations with abdominal discomfort and length of clinical signs. Abdominal discomfort was not associated with surgical complexity; however, a longer duration of clinical signs was associated with entry into the gastrointestinal tract and resection‐and‐anastomosis.

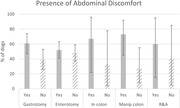

## 1 INTRODUCTION

Gastrointestinal foreign body obstructions (FBO) are considered by veterinarians to be one of the three most common causes for canine abdominal visceral pain.^1^ Clinical signs of abdominal discomfort may include gait or posture changes, physiologic changes such as hyporexia and vomiting, a tense or reactive abdomen on palpation, and changes in behaviour such as lethargy (Beal, 2005; Catanzaro et al., 2016; Wiese, 2015). These clinical manifestations of pain are often non‐specific, and a complete diagnostic work‐up is required to identify its source. Gastrointestinal FBO often present with similar clinical signs, which vary with the duration, location, and extent of the obstruction (Aronson et al., 2000).

In the case of an FBO, pain arising from the gastrointestinal system may be the result of activation of free nerve endings (A‐ δ and C‐polymodal fibre nociceptors) in the gastrointestinal wall via mechanical stretching of the intestines by the foreign body, ischemia from decreased blood flow, periods of high myoelectric activity, and inflammatory mediators secondary to gastroenteritis or necrosis (Beal, 2005; Ellison, 2010; Mazzaferro, 2003).

Abdominal discomfort has been documented to be a common clinical finding of FBO in dogs with an acute abdomen (Aronson et al., 2000; Beal, 2005; Böhmer et al., 1990; Ellison, 2010; Koike et al., 1981), and, in the authors’ personal experience, many veterinary clinicians anecdotally consider the majority of FBO to be painful. However, Hobday et al. (2014) concluded that only 44% of gastrointestinal FBO were painful, with 38% of non‐linear FBO and 55% of linear FBO exhibiting signs of abdominal discomfort. Capak et al. (2001) claimed that most cases of gastrointestinal FBO at their hospital over an 18‐year period had painful abdomens, but the authors did not report a specific percentage.

The average duration of clinical signs prior to diagnosis of an FBO has been reported to be 4–6 days (Böhmer et al., 1990; Capak et al., 2001; Hayes, 2009; Koike et al., 1981). Variability in time to presentation can be influenced by the degree of obstruction, the severity of clinical signs, and an owner's sense of urgency. An association between longer duration of clinical signs and increased mortality has previously been reported (Hayes, 2009).

Small intestinal foreign body obstructions (SIFBO) account for 67%–80% of gastrointestinal FBO (Hayes, 2009; Hobday et al., 2014). To avoid intestinal perforation, prolonged electrolyte and acid‐base imbalances, or bacterial translocation, emergency surgical removal is indicated (Aronson et al., 2000; Boag et al., 2005; Ellison, 2010; Papazoglou & Rallis, 2003). Depending on the obstruction location and subsequent bowel health, possible surgical procedures include enterotomy, intestinal resection‐and‐anastomosis (R&A), gastrotomy after manipulation of the foreign material into the stomach, or manipulation of the material into the colon for future defecation or rectal retrieval. These procedures vary in their complexity as well as overall success and financial cost. Enterotomies typically have a complication rate of 2% (Strelchik et al., 2019). R&A, a more technically challenging procedure, has a reported dehiscence rate of 13%–16% for hand‐sutured closures and 5%–11% for stapled closures (Depompeo et al., 2018; Duell et al., 2016). Gastrotomy, generally considered the preferred surgery when an incision into the gastrointestinal tract (GIT) is required, does not have a published dehiscence rate. Gastrotomy dehiscence is only listed in surgical textbooks as uncommonly occurring (Slatter, 2003). The low dehiscence rate is likely secondary to a robust blood supply promoting healing, ample tissue layers allowing for two‐layer closure, and low intraluminal pressures (Boscan et al., 2014; Johnston & Tobias, 2018). In specialty practices, there can be a significant price difference between these procedures.

Our study sought to retrospectively evaluate the potential relationships of abdominal discomfort and duration of clinical signs (prior to presentation) with the surgical procedure required to resolve canine SIFBO. Five surgical treatment groups were considered: gastrotomy after manipulation into the stomach, enterotomy, R&A, manipulated into colon, and already in the colon at the time of surgery. Secondary objectives were to assess if surgical entry into the GIT (gastrotomy, enterotomy, or R&A) was associated with the presence of abdominal discomfort on physical examination or the duration of clinical signs prior to presentation, to compare groups that required surgical entry into the GIT according to whether or not resection of a portion of the intestine was required (gastrotomy/enterotomy versus R&A), and to compare groups that required gastric versus intestinal procedures (gastrotomy versus enterotomy/R&A).

We hypothesized that dogs with obstructions necessitating intestinal R&A would have higher rates of abdominal discomfort and longer durations of clinical signs than dogs with foreign bodies that could be manipulated into the colon or into the stomach for gastrotomy. Similarly, we hypothesized that dogs with SIFBO requiring enterotomy would have an increased frequency of abdominal discomfort and longer duration of clinical signs, though less so than those with SIFBO requiring R&A.

## 2 METHODS

The medical record databases of two private practice specialty hospitals were retrospectively searched to identify dogs that had undergone exploratory laparotomy for a SIFBO confirmed via diagnostic imaging in a 1‐year period from April 2019 to April 2020. Cases with incomplete physical examinations or surgical reports were excluded. Dogs with incidental FBO discovered at surgery for other concurrent severe acute abdominal pathology—such as dystocia, gastrointestinal neoplasia, hemoabdomen, mesenteric torsion, pyometra, and septic peritonitis—were also excluded from the study.

Information obtained from the medical records included signalment, duration of clinical signs, presence of discomfort on abdominal palpation, location of the gastrointestinal foreign body, surgical procedure performed, and the nature of the foreign body. Discomfort on abdominal palpation was recorded as present if the physical examination documented pain, discomfort, a tense abdomen, splinting, or slight stylistic variations of the previously stated descriptors. The procedures performed were condensed into five groups: manipulated into the stomach for gastrotomy only, enterotomy (including cases where a gastrotomy was concurrently performed), R&A (including cases where an enterotomy or gastrotomy was concurrently performed), manipulated into the colon, and already in the colon.

Distributions of continuous variables (age, weight, number of days that clinical signs were present prior to presentation) within each treatment group were assessed by visualizing Q‐Q plots and performing Shapiro–Wilk tests for normality. Each variable failed multiple tests for normality within at least one treatment group. Various data transformations were evaluated, including logarithms and Box‐Cox transformations; however, none of the transformations satisfied criteria for a normal distribution. Therefore, each continuous variable was analyzed using the nonparametric Kruskal–Wallis test. If the *p*‐value from the Kruskal–Wallis was less than 0.05, pairwise comparisons were performed using Wilcoxon rank sum tests and Hochberg's step‐up procedure for multiple comparisons. Categorical variables (sex and presence of abdominal discomfort) were compared among treatment groups using Fisher's exact tests.

Kruskal–Wallis tests, Wilcoxon rank sum tests, and Fisher's exact tests for 2 × 2 tables were performed using Prism v9. Fisher's exact tests for 5 × 2 tables were performed (www.quantitativeskills.com/sisa/statistics/fiveby2.htm).

## 3 RESULTS

### 3.1 Animals

Over the 1‐year period, 186 dogs from the two institutions satisfied the inclusion criteria. Five dogs were excluded due to an incomplete medial record (*n* = 3) or concurrent severe acute abdominal pathology (*n* = 3), with one patient having both incomplete records and concurrent pathology.

The most common breeds among the 181 evaluable dogs were Labrador Retriever (*n* = 22, 12.2%), Golden Retriever (*n* = 18, 9.9%), Boxer (*n* = 5, 2.8%), Doberman (*n* = 5, 2.8%), French Bulldog (*n* = 5, 2.8%), and American Bulldog (*n* = 5, 2.8%). Mixed breed dogs accounted for 31.5% (*n* = 57). The median age was 4 years (range 3 months to 14 years) (Table 1). The sex distribution was 100 male castrated (55.2%), 48 female spayed (26.5%), 30 male intact (16.5%), and 3/181 female intact (1.7%) (Table 2).

**TABLE 1:** Age and weight characteristics

Variable	Group	N	Median	Min	Max	Kruskal–Wallis *p*‐value
Age (years)	Gastrotomy, manipulated in	54	4.5	0.58	14	0.39
	Enterotomy	84	5	0.25	14	
	R&A	22	4	0.42	13	
	Manipulated into colon	15	4	0.42	12	
	In colon	6	1	0.41	7	
	Total	181	4	0.25	14	
Weight (kg)	Gastrotomy, manipulated in	54	24.5	6.1	56	0.49
	Enterotomy	84	23.25	2.3	64	
	R&A	22	24.6	7.8	40.4	
	Manipulated into colon	15	18.9	8.8	40.4	
	In colon	6	25.2	10.8	42	
	Total	181	24.4	2.3	64	

Abbreviation: R&A, resection‐and‐anastomosis.

*p* < 0.05 = significant.

**TABLE 2:** Sex characteristics

	Sex				
Procedure	FI	FS	MI	MC	Total
Gastrotomy, manipulated in	2 (3.7%)	18 (33.3%)	6 (11.1%)	28 (51.9%)	54 (100%)
Enterotomy	1 (1.2%)	21 (25.0%)	17 (20.2%)	45 (53.6%)	84 (100%)
R&A	0 (0.0%)	5 (22.7%)	4 (18.2%)	13 (59.1%)	22 (100%)
Manipulated into colon	0 (0.0%)	4 (26.7%)	1 (6.7%)	10 (66.7%)	15 (100%)
In colon	0 (0.0%)	0 (0.0%)	2 (33.3%)	4 (66.7%)	6 (100%)
Total	3 (1.7%)	48 (26.5%)	30 (16.6%)	100 (55.2%)	181 (100%)
	p‐value (Fisher's exact test) = 0.68				

Abbreviation: R&A, resection‐and‐anastomosis.

*p* < 0.05 = significant.

### 3.2 Medical history and physical examination

At the time of initial physical examination at the two referral hospitals, the dogs had a mean body weight of 24.4 kg (range, 2.3–64) (Table 1). Abdominal discomfort was noted in 107 of the dogs (59%). Additional clinical signs included vomiting (98%), hyporexia (68%), lethargy (61%), and diarrhoea (15%) (Table 3).

**TABLE 3:** Clinical signs at presentation

Procedure	Vomiting	Percentage (95% CI)
Gastrotomy	54/54	100% (93.4%–100%)
Enterotomy	83/84	98.8% (93.5%–100%)
R&A	21/22	95.5% (77.2%–99.9%)
Manipulated into colon	14/15	93.3% (68.1%–99.8%)
In colon	6/6	100% (54.1%–100%)
Total	178/181	98.3% (95.2%–99.7%)
	Fisher's exact test *p*‐value = 0.25	

**Procedure**	**Hyporexia**	**Percentage (95% CI)**
Gastrotomy	34/54	63.0% (48.7%–75.7%)
Enterotomy	60/84	71.4% (60.5%–80.8%)
R&A	17/22	77.3% (54.6%–92.2%)
Manipulated into colon	9/15	60.0% (32.3%–83.7%)
In colon	3/6	50.0% (11.8%–88.2%)
Total	123/181	68.0% (60.6%–74.7%)
	Fisher's exact test *p*‐value = 0.50	

**Procedure**	**Lethargy**	**Percentage (95% CI)**
Gastrotomy	35/54	64.8% (50.6%–77.3%)
Enterotomy	51/84	60.7% (49.5%–71.2%)
R&A	14/22	63.6% (40.7%–82.8%)
Manipulated into colon	9/15	60.0% (32.3%–83.7%)
In colon	1/6	16.7% (0.4%–64.1%)
Total	110/181	60.8% (2.7%–9.9%)
	Fisher's exact test *p*‐value = 0.29	

**Procedure**	**Diarrhoea**	**Percentage (95% CI)**
Gastrotomy	7/54	13.0% (5.4%–24.9%)
Enterotomy	11/84	13.1% (6.7%–22.2%)
R&A	4/22	18.2% (5.2%–40.3%)
Manipulated into colon	3/15	20.0% (4.3%–48.1%)
In colon	2/6	33.3% (4.3%–77.8%)
Total	27/181	14.9% (10.1%–21.0%)
	Fisher's exact test *p*‐value = 0.53	

Abbreviations: CI, confidence interval; R&A, resection‐and‐anastomosis.

*p* < 0.05 = significant.

### 3.3 Surgical outcome

All 181 dogs underwent laparotomy on an emergent basis after diagnostic imaging confirmation of a SIFBO. Surgery was performed by multiple board‐certified surgeons and surgical residents. All dogs in this study survived to the time of discharge. The surgical procedure included gastrotomy after manipulation of the foreign body into the stomach in 54 (29.8%, two had additional foreign bodies manipulated into colon), enterotomy in 84 (46.3%, 18 with concurrent gastrotomy), intestinal R&A in 22 (12.2%, four with concurrent gastrotomy, two with concurrent enterotomy, and one with concurrent gastrotomy and enterotomy), manipulation of the foreign body into the colon in 15 (8.3%), and the foreign body already in the colon at surgery in six (3.3%). When the surgeries requiring entry into the gastrointestinal tract were condensed into surgical groups related to the most technically challenging portion, the surgical groups included enterotomy in 82 (45.3%), gastrotomy after manipulation of the foreign body into the stomach in 54 (29.8%), and R&A in 22 (12.2%) dogs.

Of the 107 dogs that had abdominal discomfort at initial presentation, the surgical procedure performed was enterotomy in 48 (44.9%) dogs with a concurrent gastrotomy in 13 of these cases, gastrotomy after manipulation of the foreign body into the stomach in 32 (29.9%) dogs, R&A in 16 (15%) dogs with a concurrent enterotomy or gastrotomy in six of these cases, manipulation of the foreign body into the colon in nine (8.4%) dogs, and the foreign body already in the colon at surgery in two (1.9%) dogs.

At surgery, discrete foreign bodies were found in 131/181 (72.3%), while linear foreign bodies were noted in 50/181 (27.6%). Discrete foreign bodies were located in the jejunum for 100/131 (76.3%), duodenum for 14/131 (10.7%), stomach for 11/131 (8.4%), ileum for 10/131 (7.6%), and colon for 9/131 (6.9%). Linear foreign bodies were located extending from the oral cavity to jejunum in 1/50 (2%), stomach to duodenum in 10/50 (20%), stomach to jejunum in 28/50 (56%), stomach to colon in 4/50 (8%), linear within the jejunum in 4/50 (8%), and duodenum to jejunum in 3/50 (6%). For linear FBO, 32/50 (64%) were uncomfortable. For discrete FBO, 72/131 (55%) were uncomfortable.

The nature of the foreign body was reported in 147/181 cases with the most common foreign bodies being unidentified fabric (*n* = 23), unidentified plastic (*n* = 15), corncob (*n* = 13), ball (*n* = 11), sock (*n* = 10), acorn (*n* = 6), string (*n* = 6), and toy squeaker devices (*n* = 6).

### 3.4 Group comparisons

**FIGURE 1:**
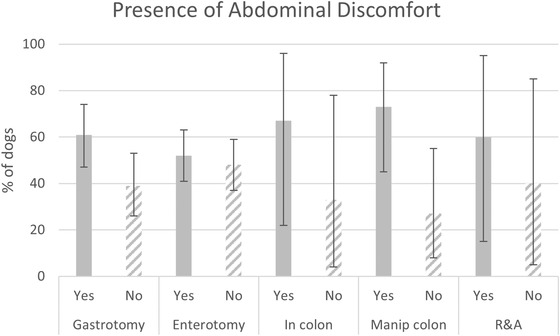
Presence of abdominal discomfort. Standard error bars reported as 95% confidence interval.

When patients were then grouped by procedure (enterotomy, gastrotomy, in colon, manipulated into colon, R&A), there were no statistically significant differences among the groups with respect to age (*p* = 0.39; Table 1), weight (*p* = 0.50; Table 1) sex (*p* = 0.68; Table 2), abdominal discomfort (*p* = 0.49; Figure 1), or any other clinical signs (Table 3). The duration of clinical signs prior to presentation was significantly different among procedures (*p* = 0.0065; Table 4). Multiple pairwise comparisons revealed that the duration was significantly longer for R&A (median = 3 days) compared with manipulated into colon (median = 1 day), gastrotomy (median = 2 days), and in colon (median 1.5 days), *p* < 0.05 for each comparison.

**TABLE 4:** Number of days clinical signs present prior to presentation

Procedure	No. of dogs	Median	Range	Wilcoxon rank sum test *p*‐value
Gastrotomy, manipulated in	54	2	1–6	0.0065
Enterotomy	84	2	1–14	
R&A	22	3	1–9	
Manipulated into colon	15	1	1–7	
In colon	6	1.5	1–2	
Total	181	2	1–14	

*Note*: Pairwise multiple comparisons using a Hochberg step‐up procedure indicated that the number of days that clinical signs were present prior to presentation was statistically significantly greater for the R&A group compared to Manipulated into colon, in colon, and gastrotomy groups, *p* < 0.05.

**FIGURE 2:**
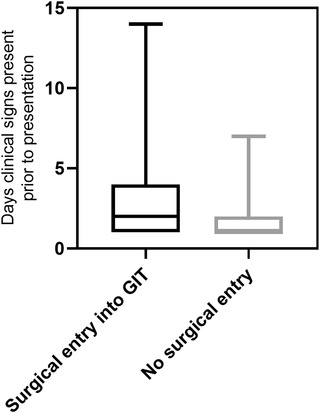
Box and whisker plot for number of days clinical signs present prior to presentation for dogs where surgical entry into gastrointestinal tract (GIT) required versus not required. Boxes represent the interquartile range from the 25th to 75th percentile with the median value indicated by the black horizontal line within the boxed portion. The bars show the range of the group data.

When patients were then grouped according to whether they required surgical entry into their GIT (gastrotomy, enterotomy, or R&A) versus not required (manipulated into the colon or in the colon), there were no significant differences in regard to the presence of abdominal discomfort (*p* = 0.64, Table 5); however, there was a significant difference for the number of days that clinical signs were present prior to presentation (median = 2 and 1, respectively, *p* = 0.0083) (Figure 2).

**TABLE 5:** Comparisons of combined groups

Procedure	Abdominal discomfort	Percentage (95% CI)	Fisher's exact test *p*‐value
Gastrotomy/enterotomy/R&A	96/260	60.0% (52.0%–67.7%)	0.49
Manipulated into colon/in colon	11/21	52.4% (28.8%–74.3%)	
Total	107/181	59.1% (51.6%–66.4%)	
Gastrotomy/enterotomy	80/138	58.0% (49.3%–66.3%)	0.24
R&A	16/22	72.7% (49.8%–89.3%)	
Total	96/160	60.0% (52.0%–67.7%)	
Gastrotomy	32/54	59.3% (45.0%–72.4%)	1.00
R&A	64/106	60.4% (50.4%–69.7%)	
Total	96/160	60.0% (52.0%–67.7%)	

Abbreviations: CI, confidence interval; R&A, resection‐and‐anastomosis.

*p* < 0.05 = significant.

**FIGURE 3:**
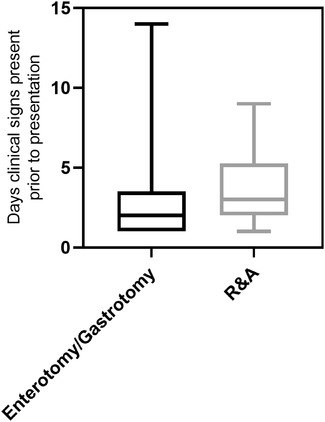
Box and whisker plot for number of days clinical signs present prior to presentation for dogs that had gastrotomy/enterotomy versus resection‐and‐anastomosis (R&A). Boxes represent the interquartile range from the 25th to 75th percentile with the median value indicated by the black horizontal line within the boxed portion. The bars show the range of the group data.

For the patients that required surgical entry into their GIT without R&A and those that required R&A, there were no significant differences in regard to the presence of abdominal discomfort (*p* = 0.24); however, there was a significant difference for the number of days that clinical signs were present prior to presentation (median = 2 and 3, respectively, *p* = 0.0076) (Table 5, Figure 3).

**FIGURE 4:**
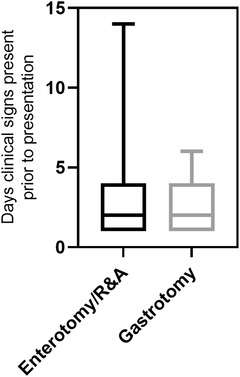
Box and whisker plot for number of days clinical signs present prior to presentation for dogs that had gastrotomy versus enterotomy/resection‐and‐anastomosis (R&A). Boxes represent the interquartile range from the 25th to 75th percentile with the median value indicated by the black horizontal line within the boxed portion. The bars show the range of the group data.

For the patients that were grouped according to whether they had gastrotomy versus intestinal surgery (enterotomy and R&A), there were no significant differences in regard to the presence of abdominal discomfort (*p* > 0.99) or the number of days that clinical signs were present prior to presentation (median = 2 and 2, respectively, *p* = 0.28) (Table 5, Figure 4).

## 4 DISCUSSION

Patient history and documentation of clinical signs are vital to the work‐up of canine acute abdomens and the decision to pursue diagnostic imaging for FBO diagnosis. For primary care veterinarians and emergency clinicians, surgical statistics are invaluable when discussing FBO with clients to provide realistic expectations for the surgical procedure to be performed, estimated cost, and overall prognosis. A subsection of primary care veterinarians is comfortable with performing abdominal exploratory surgery; however, with the increased availability of specialty surgery practices, primary care veterinarians may be more likely to refer a case if preoperative factors indicate a higher likelihood of surgical complexity, prolonged recovery, or overall poorer prognosis. Additionally, in cases of financial constraint, preoperative prognostic indicators can be helpful in deciding between euthanasia and surgical exploration.

Previous reports have shown the preoperative significance of longer clinical sign duration, hypoalbuminemia, increased serum lactate, higher American Society of Anaesthesiologists physical status classification, and septic peritonitis on survival and intestinal dehiscence rates (Hayes, 2009; Davis et al., 2017; Gill et al., 2019; Ralphs et al., 2003); however, the presence of abdominal discomfort and the duration of clinical signs have not been previously studied in relationship to the surgical procedure required for resolution of SIFBO.

In our population of 104/181 (57.5%) SIFBO cases that had abdominal discomfort, the presence of discomfort was not significantly associated with the necessity of small intestinal versus gastric surgery, the need for intestinal resection, or even of entry into the GIT. The overall rate of discomfort noted in this population of patients was higher than Hobday et al.’s (2014) previous report of 44% of all gastrointestinal FBO. SIFBO have additional routes for visceral pain via mechanical stretching of the intestinal wall and subsequent ischaemia (Beal, 2005; Ellison, 2010; Mazzaferro, 2003), whereas pain associated with a gastric foreign body is likely secondary only to inflammatory mediators from gastroenteritis. Similar to the results of Hobday et al. (2014), a higher percentage of linear foreign body cases were painful versus the percentage of discrete FBO cases (55% vs. 38%; 64% vs. 55%, respectively). This difference is likely secondary to a larger portion of the bowel being involved in the obstruction, as well as the linear material traumatizing the intestinal lumen as it becomes taut on the mesenteric side secondary to peristalsis. It should be noted that analysis of both the nature of the foreign body and the location of linear foreign body anchor and termination was beyond the scope of this study.

Maxwell et al. (2021) previously examined the effect of delayed surgical treatment on the outcomes of gastrointestinal FBO and determined that outcome did not differ; however, a higher likelihood of R&A, increased duration of surgery, longer hospitalization, and later return to eating were all associated with delayed surgical intervention. While the exact timing from hospital admission to surgical procedure was unable to be ascertained from the medical records of all cases, and was therefore beyond the scope of our study, the duration of clinical signs prior to surgery was similarly associated with a higher rate of entry into the GIT. Additionally, a longer course of clinical signs was noted in R&A cases when compared to those manipulated into the stomach for gastrotomy, manipulated into the colon, and in colon groups. However, given that the range in mean duration of clinical signs was only 2 days between groups, this difference is unlikely to be clinically relevant. While potentially useful to set owner expectations, these findings should not be used in deciding whether to pursue surgery. While all cases in our study survived to the time of discharge, Hayes (2009) reported that a longer timespan from initiation of clinical signs to surgical intervention was associated with increased mortality. As a result, a detailed history, with particular attention to the duration of clinical signs, should be obtained from the owner in cases of suspected SIFBO.

In our study, the mean duration of clinical signs was 2.7 days, a shorter timeframe than the previously reported 4–6 days for gastrointestinal foreign bodies (Böhmer et al., 1990; Capak et al., 2001; Hayes, 2009; Koike et al., 1981). Given our focus on only SIFBO, the mean duration may have been shorter due to SIFBOs causing an acute, complete occlusion of the lumen. Previous analyses included all gastrointestinal foreign bodies, and non‐occlusive gastric foreign bodies may have presented later due to less severe clinical signs. Variability in time to presentation could also have been biased by our owner population's overall sense of urgency compared to the populations in other studies over a decade prior and in different countries.

Several limitations were present within this study. Due to its retrospective nature, the obtainment of clinical history was not a standardized process. Owners may have exhibited recall bias regarding the length of clinical signs. Initial physical examinations were performed by multiple veterinarians (68 in total) with varying levels of clinical experience, and the delineation of abdominal discomfort was subjective and not determined via a verified pain scale such as the Glasgow Composite Measures of Pain Scale. Additionally, omission bias may be present if clinicians failed to document abdominal discomfort findings. Unfortunately, the subjective nature of determining abdominal discomfort is widespread in veterinary medicine, with 74%–80% of veterinarians not utilizing a specific pain score during physical examination (Catanzaro et al., 2016; Weber et al., 2012). Each published pain score also has limitations and can be considered inefficient or unrealistic in certain clinical settings (Hansen, 2003; Sharkey, 2013). However, generalized abdominal discomfort is routinely noted on physical examination of patients experiencing gastrointestinal upset, as in our study population (Beal, 2005; Catanzaro et al., 2016; Wiese, 2015). Finally, the determination of intestinal tissue viability prior to resection and anastomosis was not standardized between the multiple surgeons, and some surgeons may have performed a R&A when others would have performed an enterotomy. Recommended evaluation of intestinal viability includes intestinal colouration, presence of pulses, and continued peristalsis (Ellison, 2010). Selection bias may have been present due to the incompleteness of the medical record or improper classification of cases in the electronic medical record.

In conclusion, abdominal discomfort is not a significant indicator of associated surgical complexity and risk; however, the duration of clinical signs is an additional preoperative factor to consider in canine SIFBO cases along with the previously recommended factors of cardiovascular stability, serum lactate, electrolyte and acid/base imbalances, septic peritonitis, and overall anaesthetic risk. Additional studies using a verified pain scale and further standardization of intestinal viability parameters are needed to evaluate the relationship between abdominal discomfort and the degree of surgical intervention required.

## AUTHOR CONTRIBUTIONS

Schoelkopf: Conceptualization, data curation, methodology, project administration, writing‐original draft preparation, writing‐review & editing. Stewart: formal analysis, funding aquisition, supervision, visualization, writing‐review & editing. Sue A. Casale: Project administration, supervision, and writing‐review and editing. Fryer: project administration, supervision, writing‐review & editing.

## CONFLICT OF INTEREST

SD Stewart and KJ Fryer are employed by Ethos Veterinary Health. AC Schoelkopf is a former employee of Ethos Veterinary Health.

### ETHICS STATEMENT

All legal and ethical requirements have been met with regards to the humane treatment of animals in this study.

### PEER REVIEW

The peer review history for this article is available at https://publons.com/publon/10.1002/vms3.1045.

## Data Availability

The data that support the findings of this study are available from the corresponding author upon reasonable request.
